# Autonomous Searching for a Diffusive Source Based on Minimizing the Combination of Entropy and Potential Energy

**DOI:** 10.3390/s19112465

**Published:** 2019-05-29

**Authors:** Cheng Song, Yuyao He, Xiaokang Lei

**Affiliations:** 1 School of Marine Science and Technology, Northwestern Polytechnical University, Xi’an 710072, China; songcheng@mail.nwpu.edu.cn; 2 School of Information and Control Engineering, Xi’an University of Architecture and Technology, Xi’an 710055, China; leixiaokang@mail.xjtu.edu.cn; 3 MOE KLINNS Lab, Xi’an Jiaotong University, Xi’an 710049, China

**Keywords:** mobile sensor, infotaxis, exploration-exploitation, free energy

## Abstract

The infotaxis scheme is a search strategy for a diffusive source, where the sensor platform is driven to reduce the uncertainty about the source through climbing the information gradient. The infotaxis scheme has been successfully applied in many source searching tasks and has demonstrated fast and stable searching capabilities. However, the infotaxis scheme focuses on gathering information to reduce the uncertainty down to zero, rather than chasing the most probable estimated source when a reliable estimation is obtained. This leads the sensor to spend more time exploring the space and yields a longer search path. In this paper, from the context of exploration-exploitation balance, a novel search scheme based on minimizing free energy that combines the entropy and the potential energy is proposed. The term entropy is implemented as the exploration to gather more information. The term potential energy, leveraging the distance to the estimated sources, is implemented as the exploitation to reinforce the chasing behavior with the receding of the uncertainty. It results in a faster effective search strategy by which the sensor determines its actions by minimizing the free energy rather than only the entropy in traditional infotaxis. Simulations of the source search task based on the computational plume verify the efficiency of the proposed strategy, achieving a shorter mean search time.

## 1. Introduction

Autonomous robots carrying appropriate sensors can be deployed to efficiently localize the source of a biochemical or radiological contaminant leakage, such as an oil spill or a radioactive dispersal, and track the contaminant dispersion in turbulent flows [1,2]. This issue of source search, referred to odor or gas source localization, has received considerable research in recent years [3,4,5,6]. In general, variations in material concentrations from a source in a flow field are heavily dependent on the Reynolds numbers. Gradient-based strategies, such as extremum seeking [7], *Escherichia coli* algorithms [8], and Braitenberg algorithms [9], work well in a low Reynolds environment with smooth variations in material concentrations. However, in a turbulent environment with high Reynolds, the dispersion from a source is typically broken into unsteady, sparse, and disconnected patches [10,11]. It results in a sporadic and intermittent sensory landscape, with fluctuating variations without the gradient pointing towards the source [12], rendering the gradient-based strategies ineffective or even invalid [13]. This work focuses on the search for a diffusive source of unknown location in the open wind field where turbulence can cause irregular gradients and intermittent sensory cues.

The search problem in a turbulent environment can be formulated as a probabilistic search to account for stochastic intermittent detections. A class of probabilistic search strategies referred to as infotaxis [14] is used specifically for seeking the diffusive source in a turbulent medium, which determines actions to reduce the uncertainty about the source through minimizing the entropy of the source probability distribution. The infotaxis scheme has been effectively exploited and developed for many search strategies. Masson [15] proposed an infotaxis scheme termed mapless, allowing the search in complex varying environments with limited space perception based on the minimization of free energy. Ristic et al. [16] investigated the performances of an infotaxis scheme based on three different reward functions, developing an improved infotaxis scheme based on Rényi divergence as well. Hutchinson et al. [17] developed the entrotaxis scheme that drives the searcher to the position of the most uncertainty in the next detection, instead of the position of the minimum uncertainty in the expected posterior source distribution. Mishra et al. [18] proposed the expected rate algorithm and proved that both infotaxis and expected rate algorithms generate identical optimization steps in most cases.

The exploration-exploitation balance is the key to maintain the search efficiency leveraging these stochastic detections [19]. For the infotaxis method, the expected reduction of the entropy is implemented as the exploration term (that is, gathering more information and obtaining a more reliable estimate of the source distribution) and the maximum likelihood as the exploitation term (that is, going to the estimated most probable source location) [20]. This work addresses the drawback in the traditional infotaxis strategy [14] that tends to favor the exploration over exploitation of the information, resulting in search behavior with more traverse motions and spending more search time. There exists an exploitation term playing the role of the maximum likelihood. Nevertheless, it employs the local probability around the sensor for the maximum likelihood, which prevents the chasing behavior from being led off the track with the receding of uncertainty after acquiring more detections. The problem lies in that the small divergence of the local probabilities is not available to produce a significant gradient towards the most probable source. Moreover, we notice that the exploitation by directly going to the global most probable source location is very risky because the estimated probability distribution is multimodal and not reliable before obtaining adequate detections [21]. In fact, a maximum likelihood or maximum a posteriori strategy systematically fails far from the source because of the misrepresentation of the environment by the unreliable probability distribution. Thus, the balance between exploration and exploitation should be dynamically adaptive according to the degree of the probability distribution’s reliability. In this case, Masson [15] has employed a local probability with an extended domain to reinforce the maximum likelihood behavior that shifts the balance toward exploitation.

To balance exploration-exploitation and speed up the search progress, we propose a novel search scheme that minimizes the combination of entropy and potential energy, formalized as a form of free energy [15,21,22], where the mobile sensor platform determines its search action towards the minimization of the free energy. The entropy drives the sensor to accumulate the information (as in the conventional infotaxis). The potential energy, involving the weighted sum of the sensor’s distance to hypothetical sources, is added to reinforce the chasing behavior. The temperature actively controls the relative value between the potential energy and the entropy. The varying temperature is reduced by levering the trace of the covariance matrix of the probability distribution and so shifts the balance toward exploitation with the receding of the uncertainty or the increasing reliable estimation. Similar to [16,17,23], we employ a particle filter representation of the source probability distribution to make the strategy computationally tractable for large complex spaces. Then, the potential energy is computed by the spread of the particles and the distance between the current position and all the particles. We demonstrate the efficiency of the scheme numerically, with a computational model of odor plume propagation. The contribution of this paper is that free energy is introduced to replace the entropy for decision making, which shifts the exploration-exploitation balance toward exploitation with the receding of the uncertainty about the source. It can lead to a faster search for a diffusive source in a large space and result in a shorter path to reach the source for mobile sensor platforms.

The organization of the paper is as follows. The problem formulation is presented in Section 2. The scheme of free energy infotaxis is described in Section 3. Section 4 presents the numerical results, through simulations using a computational plume dataset characterized by a turbulent flow. Finally, the conclusions are drawn in Section 5.

## 2. Problem Formulation

### 2.1. Infotaxis Scheme

Infotaxis was introduced in [14] for searching in complex environments with stochastic sporadic detections. It is built around two core components: Bayesian estimation of the source position based on detection history and greedy decision making based on entropy minimization. Bayesian estimation is employed to construct the posterior probability distribution about the source location. Greedy decision making is to choose the searcher’s motion direction gathering the information reward computed on the probability distribution.

Suppose that the diffusive source is located at coordinates specified by $r_{0}={\left(x_{0},y_{0}\right)}^{T}\in W$, where $W\in R^{2}$ denotes a free two-dimensional search area. A spherical detecting sensor with radius *a* is mounted on the mobile sensor platform, whose position is r=(x,y). The status of detection is identified as a binary variable h∈{0,1} by a sensor: h=0 indicates no dispersion at the current position of the sensor, and h=1 indicates otherwise. The counting positive detections z=sum(h) during the time interval Δt at any location *r* are modeled by the Poisson distribution as follows:

(1) $$z∼p\left(z\right)=\frac{{\left[R\left(r,r_{0}\right)\Delta t\right]}^{z}}{z!}exp\left[-(R\left(r,r_{0}\right)\Delta t\right]$$

where $R(r,r_{0})\Delta t$ denotes the expectation of positive detections in time interval Δt. The mean rate $R(r,r_{0})$ is defined as the expected number of encountering the dispersion at the given position *r* with respect to the source located at $r_{0}$. The mean rate is related to the distance from the source, the strength of the source, the dynamics of the flow field, and the geometric structure of the environment. The parameters of $R(r,r_{0})$ including strength, wind velocity and direction, and diffusivity are generally assumed to be the prior knowledge.

The detection events along the search trajectory carry the cues about the relative location of the source with respect to the sensor. We assume $d_{k}=\left(r_{k},z_{k}\right)$ encapsulates the detection at position $r_{k}$ for $z_{k}$ encounters of the dispersions at time *k*. The posterior probability $P_{k}\left(r_{0}\right)$ for the unknown position of the source utilizing Bayesian inference reads:

(2) $$\begin{array}{c} P_{k}\left(r_{0}\right)=\frac{P_{k-1}\left(r_{0}\right)\ell \left(d_{k}|r_{0}\right)}{\int_{W}P_{k-1}\left(r_{0}\right)\ell \left(d_{k}|r_{0}\right)dr_{0}} \end{array}$$

where $\ell \left(d_{k}|r_{0}\right)=p\left(z_{k},R\left(r_{k},r_{0}\right)\right)$ denotes the likelihood of the detection $d_{k}$ conditioned on the source at $r_{0}$.

In the context of information theory, the purpose of the sensor is to reduce the uncertainty of the target through the interaction with the environments. Shannon entropy is introduced to measure the uncertainty $S_{k}=-\int_{W}P_{k}\left(r_{0}\right)\log P_{k}\left(r_{0}\right)dr_{0}$. New detections can reduce the entropy and increase the amount of information. The expected change in information results from any detection or non-detection upon moving to one of the admissible locations $r_{m}$ as follows:

(3) $$\begin{array}{c} \Delta E_{S}\left(r_{k}\to r_{m}\right)=P_{k}\left(r_{m}\right)\left(0-S_{k}\right)+\left(1-P_{k}\left(r_{m}\right)\right)\sum_{\eta =0}^{\infty }\rho_{\eta }\Delta S_{\eta } \end{array}$$

where $\Delta S_{\eta }$ is the change in the entropy of the estimation if the sensor receives η={1,2,3,⋯} new positive sensor detections at the next step as it moves to the neighboring position. $\rho_{\eta }$ denotes the probability of η hits by the Poisson model. The first term on the right side corresponds to expected change in entropy upon finding the source at $r_{m}$, and the second term accounts for the case when the source is not at $r_{m}$. The targeted minimization of entropy drives the sensor to move in the direction of the most entropy drop. When the entropy is reduced to zero, the uncertainty disappears, and the source is found.

### 2.2. Deficiency in Infotaxis Scheme

The first term on the right-hand side of Equation (3) is the exploitative term, favoring motion to maximum likelihood points. The second term on the right-hand side of Equation (3) is the explorative term, favoring information gain to receive additional detections. Thus, it can be explicitly seen that the infotaxis scheme naturally combines exploitative and explorative tendencies.

The drawback presented in the infotaxis scheme is that the exploitative term only works near the end of the search. While the probability converges to the source, the searcher’s position is still far away from the source because of sensing the far field via the hit rate. This leads to the searcher locating in the zone of low probability, which cannot produce a significant gradient pointing towards the most probable position. The values of $P_{k}\left(r_{m}\right)$ for all admissible neighboring locations $r_{0}$ are small (as shown in Section 4.1). It weakens the role of exploitation played by $P_{k}\left(r_{m}\right)\left(0-S_{k}\right)$ and consistently shifts the balance of exploration-exploitation towards exploration during the search process. The sensor enters into the zone of high probability only close to the source. Subsequently, the maximum likelihood explicitly points toward the source and preforms its function at this time.

It should be noted that the probability distribution of the source is generated from the remote estimation. As a result, the sensor always lays behind the convergence rate of the probability distribution. Instead of maximum likelihood by $P_{k}\left(r_{m}\right)\left(0-S_{k}\right)$, chasing the global most probable source can lead to very efficient searches. Nevertheless, directly chasing the peak position of probability systematically fails because of the multimodal probability distribution. Moreover, strengthening the exploitation before obtaining a more reliable estimation frequently leads to a self-trap (over-exploitation). In fact, the mobile sensor platform should gradually favor the chasing behavior, where the exploitation has more influence on the decision process with the improving reliability of the probability distribution. In general, the problem is formulated as the requirement of the infotaxis scheme where the exploration and the exploitation are combined and actively balanced during the search process.

## 3. Free Energy Infotaxis Search Scheme

The details of the proposed free energy infotaxis scheme for improving the search are presented in this section. We first present the construction of free energy in the context of thermodynamic theory. Next, the particular design based on the particle filter and the computational form of POMDP (Partially-Observable Markov Decision Process) by minimizing free energy are provided.

### 3.1. Construction of Free Energy

The entropy continues to be effective as the exploration term (as in the traditional infotaxis), i.e., driving the sensor to gather information to improve the accuracy of estimation. Meanwhile, another new exploitation term that involves the attraction of the most probable source is presented with the purpose to reinforce the behavior of chasing the most probable source.

In this work, the attraction function is defined as potential energy related to the weighted sum of the distance between the current location $r_{k}$ and all the hypothetical sources $r_{0}$ with different weights expressed by the probability distribution. It avoids directly using the peak location of probability distribution $P_{k}\left(r_{0}\right)$ as the most probable source because of the multimodal nature of the probability distribution. The potential energy $W_{k}$ is defined as:

(4) $$W_{k}=\int_{r_{0}\in W}P_{k}\left(r_{0}\right)\left|\right|r_{k}-r_{0}{\left|\right|}^{\gamma }dr_{0}$$

where $\left|\right|r_{k}-r_{0}\left|\right|$ is the distance between the current location $r_{k}$ and a hypothetical source $r_{0}$ and γ is the exponent of the distance that determines the attraction strength by the hypothetical source. The probability $P_{k}\left(r_{0}\right)$ play the role of the weight of the attraction from the hypothetical source at the location $r_{0}$. The potential energy $W_{k}$ describes the synthesized attraction of all the hypothetical sources whose probability is continuously updated while acquiring new detections. This term is different from the “work energy” of the free energy in [15], which depends on the gradient in the probability map.

The combination of the entropy as exploration and the potential energy as exploitation formalizes the form of free energy. Hence, instead of the entropy in the infotaxis scheme, the free energy to be minimized reads:

(5) $$\begin{array}{cc} F_{k} & =W_{k}+TS_{k}\\ & =\int_{r_{0}\in W}P_{k}\left(r_{0}\right)\left|\right|r_{k}-r_{0}{\left|\right|}^{\gamma }dr_{0}-\alpha \cdot tr{\left(\Sigma \right)}^{\beta }\int_{r_{0}\in W}P_{k}\left(r_{0}\right)\log P_{k}\left(r_{0}\right)dr_{0} \end{array}$$

where $W_{k}$ is the potential energy and $S_{k}$ is the Shannon entropy, while $T=\alpha \cdot tr{\left(\Sigma \right)}^{\beta }$ is the temperature that controls the relative value between the two previous terms. tr(Σ) is the trace of the covariance matrix Σ of probability distribution $P_{k}\left(r_{0}\right)$, and α is a factor of proportionality, while β denotes its exponent that determines the descending rate. The value of tr(Σ) declines as the probability $P_{k}\left(r_{0}\right)$ contracts from the initial uniform distribution to the gathering distribution on the source, which indicates the reduction of the uncertainty and a more reliable estimation of the source distribution. In particular, the proportion of potential energy in free energy is adjusted by the reduction of temperature. By comparison, the temperature of free energy is kept constant in [15,22], and the proposal of varying temperature was mentioned in [15]. The reducing temperature avoids the over-exploitation of moving toward the most probable source location for the high uncertainty of the environment or low reliable probability distribution.

During the search, the term $S_{k}$ drives the sensor to accumulate the information for the increasing reliability of the estimation and reduce the uncertainty about the source. With the reduction of the uncertainty (decreasing tr(Σ)), the term $W_{k}$ gradually leads off the search and drives the sensor to chase the estimated most probable source location. Therefore, the balance is shifted from exploration ($S_{k}$) to exploitation ($W_{k}$) with the receding of the uncertainty (i.e., increasing reliability of the estimation).

### 3.2. Implementation Based on the Particle Filter

The processes of Bayesian estimation, decision making, and the weighted sum of distances all rely on the probability distribution, which is represented on a grid map in the traditional infotaxis scheme. However, the resolution of the grid map that covers the search area must be increased to accommodate the accuracy of the probability distribution. The large number of the grid cells presents additional challenges in computation on a sensor platform. To facilitate the computation intensity, the sequential Monte Carlo method is employed to represent the probability distribution with a limited and tractable amount of randomly-drawn particles. The use of a particle filter allows us to bound the computational burden on the sensor platform [16,23], which determines the probability distribution to cover the search area that is of interest.

Let us use the sequential Monte Carlo method to represent the posterior distribution $P_{k}\left(r_{0}\right)$ by a random set ${\left\{\left(r_{0,k}^{\left(m\right)},w_{k}^{\left(m\right)}\right)\right\}}_{m=1:M}$. Here, $r_{0,k}^{\left(m\right)}={\left(x_{0,k}^{\left(m\right)},y_{0,k}^{\left(m\right)}\right)}^{T}$ is the position of the random particle sampled from probability map $P_{k}\left(r_{0}\right)$ and $w_{k}^{\left(m\right)}$ is the associated weight. The weights are normalized, i.e., $\sum_{m=1}^{M}w_{k}^{\left(m\right)}=1$, and *M* is the number of particles. The approximation of the sensor’s source probability map can then be expressed as:

(6) $$P_{k}\left(r_{0}\right)\approx \sum_{m=1}^{M}w_{k}^{\left(m\right)}\delta \left(r_{0}-r_{0,k}^{\left(m\right)}\right)$$

where δ(·) is the Dirac delta function. By comparing with the grid-based method [14,15,22], Monte Carlo approximation has simplified the numerical solution of complicated integrals and made the representation of the probability map light.

Given the prior probability at time k−1 represented by ${\left\{\left(r_{0,k-1}^{\left(m\right)},w_{k-1}^{\left(m\right)}\right)\right\}}_{m=1:M}$, one can compute random samples ${\left\{\left(r_{0,k}^{\left(m\right)},w_{k}^{\left(m\right)}\right)\right\}}_{m=1:M}$ to approximate the posterior $P_{k}\left(r_{0}\right)$ at time *k*, using the importance sampling technique [24]. The unnormalized particle weights ${\tilde{w}}_{k}^{\left(m\right)}$ are computed using detections $d_{k}$ as follows:

(7) $${\tilde{w}}_{k}^{\left(m\right)}=w_{k-1}^{\left(m\right)}\ell \left(d_{k}|r_{0,k}^{\left(m\right)}\right)$$

The particle’s weight is subsequently normalized, $w_{k}^{\left(m\right)}={\tilde{w}}_{k}^{\left(m\right)}/\sum_{i=1}^{M}{\tilde{w}}_{k}^{\left(i\right)}$. Importance sampling is carried out sequentially for k=1,2,⋯,. In order to improve the resulting sample diversity, the resampled particles are subjected to an MCMC move step. The condition of resampling is that the effective size $M_{eff}=1/\sum_{m=1}^{M}{\left(w_{k}^{\left(m\right)}\right)}^{2}$ of the particles becomes less than a threshold.

As the probability distribution $P_{k}\left(r_{0}\right)$ is approximated by the sampled particles ${\left\{r_{0,k}^{\left(m\right)},w_{k}^{\left(m\right)}\right\}}_{m=1:M}$, the entropy can be calculated as $S_{k}=-\sum_{m=1}^{M}w_{k}^{\left(m\right)}\ln w_{k}^{\left(m\right)}$. The hypothetical sources are represented by the particles (not grid cells in [14]), i.e., each particle $r_{0,k}^{\left(m\right)}$ denotes a hypothetical source associated with a weight $w_{k}^{\left(m\right)}$. By the importance sample method and resample method, the number of particles needed in this case is substantially less than the previous grid cells. Then, the free energy based on particles can be calculated by:

(8) $$\begin{array}{cc} F_{k} & =W_{k}+TS_{k}\\ & =\sum_{m=1}^{M}w_{k}^{\left(m\right)}\left|\right|r_{k}-r_{0,k}^{\left(m\right)}{\left|\right|}^{\gamma }-\alpha \cdot tr{\left(\Sigma \right)}^{\beta }\sum_{m=1}^{M}w_{k}^{\left(m\right)}\ln w_{k}^{\left(m\right)} \end{array}$$

where the potential energy $W_{k}$ is the weighted sum of the distance between the current location $r_{k}$ and all the particles $r_{0,k}^{\left(m\right)}$ with the corresponding weight $w_{k}^{\left(m\right)}$. The trace tr(Σ) in temperature *T* is measured by the spread of the local positional particles ${\left\{r_{0,k}^{\left(m\right)},w_{k}^{\left(m\right)}\right\}}_{m=1:M}$ (Σ is the weighted covariance matrix of the particles’ distribution). Here, the level of uncertainty about the source and the reliability of the estimations is indicated by the spread of particles. With acquiring more detections, the spread of particles contracts to cover the area of the most probable source, which corresponds to the decrease of trace tr(Σ).

### 3.3. Infotaxis Decision by Minimizing Free Energy

The sensor platform at $r_{k}$ autonomously decides on the control variable $u_{k}$ using the free energy infotaxis strategy, which can be formulated as a partially-observed Markov decision process (POMDP) [16]. The elements of POMDP include the state, a set of admissible actions and a reward function. The state at time $t_{k-1}$ is the probability distribution $P_{k-1}\left(r_{0}\right)$ that specifies the sensor current knowledge about the source. Admissible actions $U_{k}$ can be formed with one or multiple steps ahead. A decision in the context of the search is the selection of a control vector $u_{k}\in U_{k}$. The reward function maps each admissible action into an expected information gain.

Based on the probability distribution represented by sampled particles ${\left\{r_{0,k}^{\left(m\right)},w_{k}^{\left(m\right)}\right\}}_{m=1:M}$, the POMDP decision is transferred to minimize the free energy rather than only the entropy $S_{k}$.

(9) $$u_{k}=\arg\max_{v\in U_{k}}\left\{F_{k-1}-E\left\{F_{k}\left[d_{k}\left(v\right)\right]\right\}\right\}$$

where $E\left\{F_{k}\left[d_{k}\left(v\right)\right]\right\}$ is the expected free energy, which is updated on the prior free energy $F_{k-1}$ with the future detection $d_{k}\left(v\right)$. E is the expectation operator. The space of admissible actions $U_{k}$ is continuous with dimensions: linear velocity *V*, angular velocity Ω, and duration of motion $T_{m}$. In order to reduce the computational burden of numerical optimization, $U_{k}$ is adopted as a discrete set. If V, O, and T denote the sets of possible discrete values of *V*, Ω, and $T_{m}$, respectively, then $U_{k}$ is the Cartesian product V×O×T (refer to [16]).

In the computation of $E\left\{F_{k}\left[d_{k}\left(v\right)\right]\right\}$, we need the future detection $d_{k}\left(v\right)=\left\{r_{k}\left(v\right),z_{k}\left(v\right)\right\}$ for the calculation of $w_{k}^{\left(m\right)}\left(v\right)$. However, the reward must be computed before the mobile sensor platform actually moves to $r_{k}\left(v\right)$ and acquires the next measurements $z_{k}\left(v\right)$. In practice, for a given position *r*, we compute the mean $\mu \left(v\right)=t_{0}\sum_{m=1}^{M}w_{k}^{\left(m\right)}R\left(r,r_{0,k}^{\left(m\right)}\right)$ and then find $z_{max}$ such that the distribution function corresponding to Poisson probability $p\left(z;\mu \left(v\right)\right)=e^{-\mu (v)}\mu {\left(v\right)}^{z}/z!$ (refer to Equation (1)) is greater than a certain threshold 1−η, where η≪1. The summation is then computed only for $z=0,1,\cdots ,z_{max}$. Thus, the two terms of free energy $F_{k}\left[d_{k}\left(v\right)\right]$ are calculated based on the particles $\left\{r_{0,k}^{\left(m\right)},w_{k}^{\left(m\right)}\left(v\right)\right\}$, the sensor future position $r_{k}\left(v\right)$, and measurements $z_{k}\left(v\right)$. The expected value $E\left\{F_{k}\left[d_{k}\left(v\right)\right]\right\}$ with respect to the probability mass function p(z;μ(v)) is:

(10) $$\begin{array}{c} E\left\{F_{k}\left[d_{k}\left(v\right)\right]\right\}=\sum_{z=0}^{z_{\max}}p\left(z;\mu_{v}\right)F_{k}\left[d_{k}\left(v\right)\right] \end{array}$$

The search continues until the global stopping criterion is satisfied, where the mobile sensor platform falls into the local area of the source location within a certain radius for declaring the source. If the distance between the sensor platform and the source is smaller than $R_{s}$, then the stopping criterion is satisfied and is given a value of one, otherwise it is zero.

The basic steps for the algorithm of free energy infotaxis scheme on the search sensor platform are summarized in Algorithm 1.

**Algorithm 1** the free energy infotaxis scheme1**Input:** sensor’s position $r_{k=0}$, particles ${\left\{\left(r_{0,k=0}^{\left(m\right)},w_{k=0}^{\left(m\right)}\right)\right\}}_{m=1:M}$4**while** “source not found” **do**5  Compute the free energy $F_{k-1}$ using Equation (8)6  Create the admissible set $U_{k}=V\times O\times T$7**for** every $v\in U_{k}$
**do**8  Compute the future sensor location $r_{k}\left(v\right)$9  Determine $z_{max}$ s.t. $\sum_{z=0}^{z_{max}}P\left(z;\mu \left(v\right)\right)>1-\eta$10  Compute the future free energy $F_{k}\left[d_{k}\left(v\right)\right]$11  Compute the expected reward $E(F_{k}\left[d_{k}\left(v\right)\right])$ using Equation (10)12
**end for**
13  Find $u_{k}$ in maximum $\left\{F_{k-1}-E\left\{F_{k}\left[d_{k}\left(v\right)\right]\right\}\right\}$14  Move to $r_{k}$ and detect the dispersion as $d_{k}$16  Update the particles ${\left\{\left(r_{0,k}^{\left(m\right)},w_{k}^{\left(m\right)}\right)\right\}}_{m=1:M}$ using Equation (7)17
**end**
18**Output:** the estimated source position ${\bar{r}}_{0}$

## 4. Simulations

Simulations of the source search task based on computational plume were established to study the effectiveness and efficiency of the proposed strategy. A typical run was first carried out to illustrate the performances of the traditional infotaxis and the proposed strategy. Then, average search performance, expressed by the mean search time and the mean distance, was estimated via Monte Carlo runs. Lastly, the effect of temperature *T* was investigated and discussed.

The following parameters (all physical quantities are arbitrary units (a.u.)) were used:

1. True source parameters: $X_{0}=-200,Y_{0}=0,Q_{0}=2$;
2. Search area: W=[−300,300]×[−150,150];
3. Motion model parameters: δ=0.25,V={1},O={−3,−2,−1,0,1,2,3}*π/180,T={1};
4. Environmental and sensor parameters: a=1, D=1, τ=400, V=0.5, $\Delta_{t}=1$;
5. Algorithm parameters: α=0.01; β=1.4; γ=3 and number of particles M=600, $M_{thd}=M/3$;
6. Local search stopping threshold: $R_{s}=3$.

### 4.1. Typical Run

**Figure 1:**
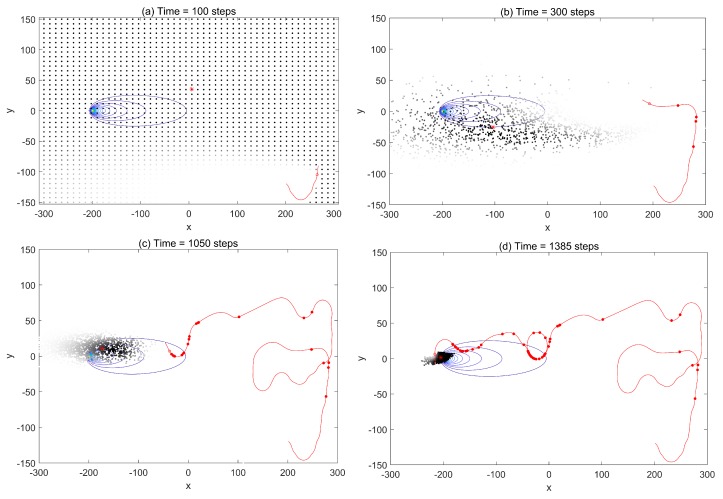
The trajectory (red line), detections (red solid circles), and particles (black dots) of the mobile sensor platform at the times *k* = 300, 500, 1080, 1385 using the infotaxis scheme. The source location at (−200,0) with the contour plot of the corresponding mean rate. The estimated source is indicated by the weighted center of the particles marked by the red star.

**Figure 2:**
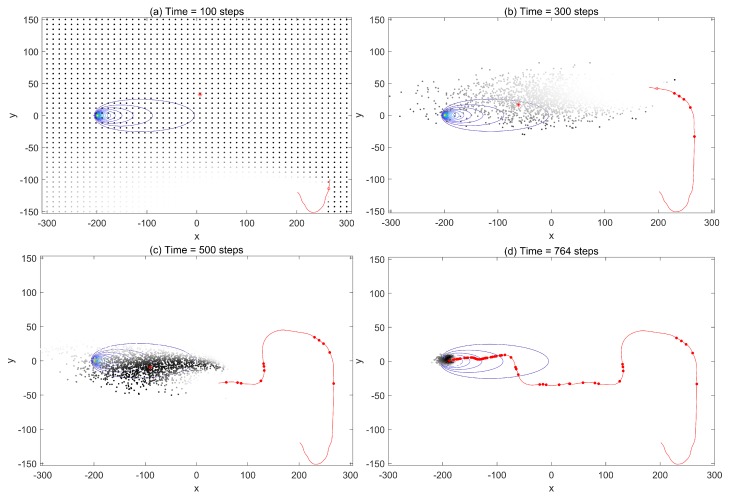
The trajectory, detections, and particles of the mobile sensor platform at the times *k* = 150, 350, 550, 764 using the free energy scheme. The source location at (−200,0) with the contour plot of the corresponding mean rate.

**Figure 3:**
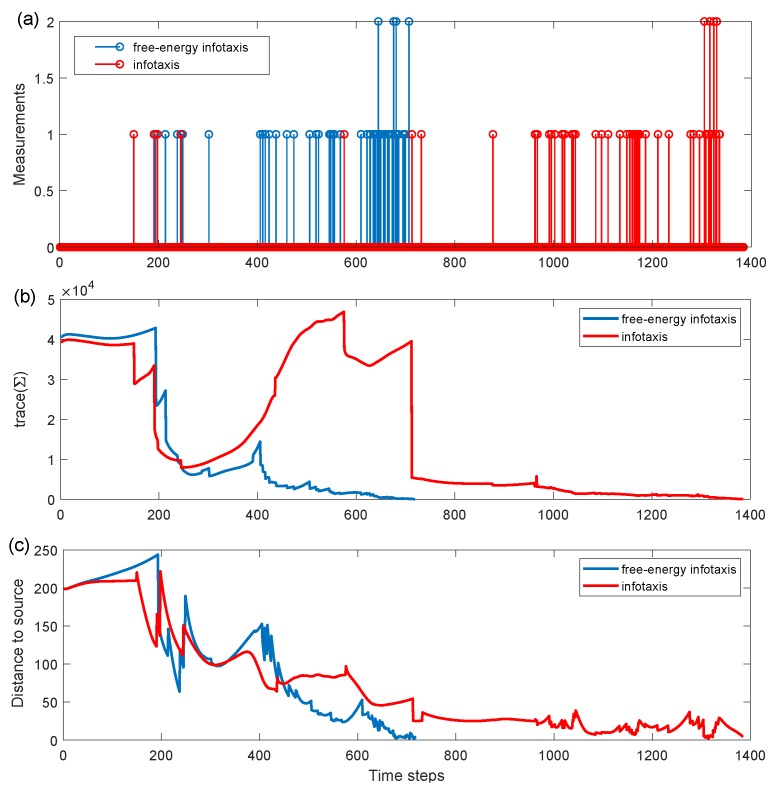
(**a**) The measurements of sensor platform over time; (**b**) the trace of the covariance matrix measuring the spread of the sampled particles; (**c**) the estimated source’s distance to the source over time, marked in red corresponding to Figure 1 and marked in blue corresponding to Figure 2.

First, we investigated the trajectories and search process to demonstrate the performances using the infotaxis scheme and the free energy infotaxis scheme, respectively. The results of typical runs on the infotaxis scheme and the free energy infotaxis scheme are shown in Figure 1 and Figure 2 respectively, and Figure 3 presents the corresponding characteristics during the search.

Figure 1 displays the search area, the trajectory of the search sensor at *k* = 100,300,1050,1385 using the infotaxis scheme, as well as the source location at (−200,0) with the contour plot of the corresponding mean rate. The random samples $r_{0,k}^{\left(m\right)}$ approximating the posterior $P_{k}\left(r_{0}\right)$ are shown as black dots. Figure 1a shows the particles before meeting the re-sampling condition, where the particles are placed on a regular grid, thus mimicking a grid-based approach, with the value of particle weights indicated by the gray-scale intensity. After acquiring the positive detections, the particles ${\left\{r_{0,k}^{\left(m\right)}\right\}}_{m=1:M}$ were resampled, and their corresponding weights were reset to the uniform 1/M (shown at k=300). At this time k=300, the spread of the sampled particles contracted, but maintained a relatively high level. This is indicated by the trace of the covariance matrix as shown in Figure 3b. Nevertheless, the mobile sensor platform tended to explore the space and generated a spiral search behavior. Then, the spread of the sampled particle contracted to a small area at k=1050 as more detections were acquired (the trace declined, as shown in Figure 3b), but the spiral search still appeared. The overall search trajectory demonstrated many turns and winds. This would cost much of the limited time of the sensor platform. The distance to the source in Figure 3c indicates the approaching rate of the sensor towards the source. In general, the expected search should be that the sensor platform targets the most estimated probable source location as the reducing spread of sample particles meets a certain level.

Figure 2 shows the search area, the trajectory of the mobile sensor platform at k=100,300,500,764 using the free energy infotaxis scheme, and its sampled particles. The trajectory is similar to that in Figure 1 before the time steps k=300, as shown in Figure 2a,b, and there were also similarities in the curves of trace tr(Σ) and the distance to the source, as shown in Figure 3b,c. As more positive detections were acquired, the spread of the particles contracted (shown at k=500), i.e., more reliable estimation or increased certainty about the source (the trace of covariance matrix declines in Figure 3b). The exploitation in the search was gradually reinforced, and the mobile sensor platform gradually tended to approach the intensive area of particles, as shown in Figure 2c. When the spread of particles contracted to a small area, the exploitation behavior led the search off track, and the sensor platform was driven to go straight to the most probable source (shown at k=764). The distance to the source shown in Figure 3c demonstrates that the chasing behavior gradually led the search off track with the improvement of the estimation and made the mobile sensor platform go straight towards the source.

**Figure 4:**
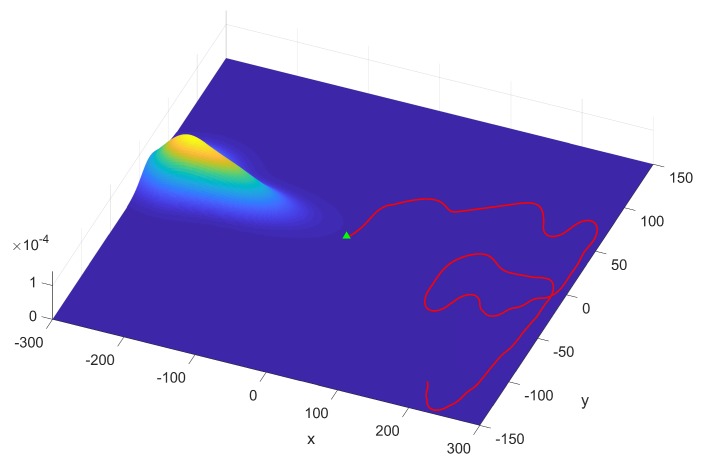
The contour plot of the probability map (the particles fitted by Gaussian Mixture Model(GMM)), the current position of the mobile sensor platform marked by a blue triangle at the time k=1020 using the infotaxis scheme.

Figure 4 is presented to show the situation that the maximum likelihood method by $P_{k}\left(r_{m}\right)\left(0-S_{k}\right)$ in the infotaxis scheme cannot effectively reinforce the exploitation via the neighboring probability or local probability. Obviously, the probability distribution contracted to cover the location of the source and reached an appropriate level of reliability (tr(Σ) declines in Figure 3b) to direct the search. However, the sensor’s position was located in a low probability area, which is unavailable to produce a significant gradient pointing towards the source. This led the term of exploitation $P_{k}\left(r_{m}\right)\left(0-S_{k}\right)$ in Equation (3) not to perform its function.

The observed results by typical runs confirmed that the availability of potential energy in the free energy infotaxis scheme is essential to improve the search performance on a given search task.

### 4.2. Monte Carlo Runs

Next, to evaluate the performance and efficiency of the proposed approach, 100 Monte Carlo runs were performed. The search was performed using the source location at the top left of the space and the initial position at the bottom right. Table 1 shows the mean search time when varying the scale of the search area, comparing the free energy infotaxis scheme with the related infotaxis schemes. These works provide improvements to the classical infotaxis method from varying perspectives. Infotaxis II [16], Infotaxis III [16], and Entrotaxis [17] perused a more effective information gain for decision making. Mapless infotaxis [15] and the proposed method based on the free energy shift the behavior of gathering information to the behavior of exploiting the information. In our simulation, we focused on the form of free energy employed by mapless infotaxis without taking incomplete space information and odometry errors into account, as in [15].

There was initially a significant increase in the mean search time for infotaxis schemes with extending the search area for exploring more place to acquire the plume. By comparison, the mean search time in the common space was shortened by the infotaxis schemes based on the free energy (mapless infotaxis and the proposed method). In particular, the proposed method with the distance potential energy and the adaptive temperature produced a slightly shorten time than the mapless infotaxis with the local probability map and constant temperature. This is because the exploitation dominated the search after obtaining a more reliable estimation. The results confirm that the proposed free energy infotaxis scheme can speed up the search progress.

**Figure 5:**
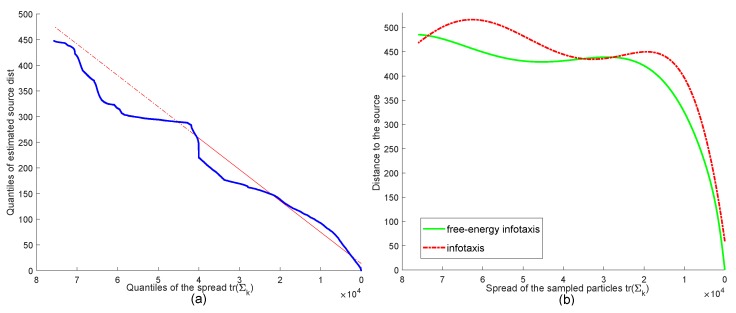
(**a**) Q-Q plot of the distance between the estimated source and the real source versus the inverse of the spread of the sampled particles. (**b**) The distance of the sensor position and the real source using the infotaxis scheme versus the free energy infotaxis scheme (curve fitting the data). The source location was fixed at [−250, 0] and the initial sensor position at [200,−100].

It should be noted that the mean search time in varying scales was almost shorter than the classical infotaxis by a uniform step (the interval 154–168), except the scale 100 × 100. This came from the fact that the acceleration of the search appeared in the phase of the exploitation. To illustrate this, Figure 5 shows the distance between the sensor and the real source, as well as the distance between the estimated source and the real source over the spread of the particles. First, the estimated source was verified to converge to the real source with the contraction of the particles, as shown in Figure 5a (the distance declined to zero with the reduction of tr(Σ)). This ensured the validity of chasing the estimated most probable source leading the sensor to the real source by the free energy infotaxis scheme. Second, with the reduction of the spread, the distance between the sensor and the source decreased, and this progress was accelerated after the spread, meeting a certain level, as shown in Figure 5b. From the comparison, the decreasing rate obtained by the free energy infotaxis scheme was faster than that of the infotaxis scheme. The results demonstrate that the sensor reinforced the behavior of going straight to the source by the free energy infotaxis scheme.

### 4.3. Effect of the Temperature *T*

Temperature *T* controls the relative value between the potential energy and the entropy, which allows active control of the exploration-exploitation balance during the search. With the reduction of uncertainty indicated by the trace tr(Σ), temperature *T* dropped, and the proportion of potential energy in free energy was strengthened, shifting the balance towards the exploitation. We ran the search simulations by setting two extreme values to investigate the effect of temperature *T*.

**Figure 6:**
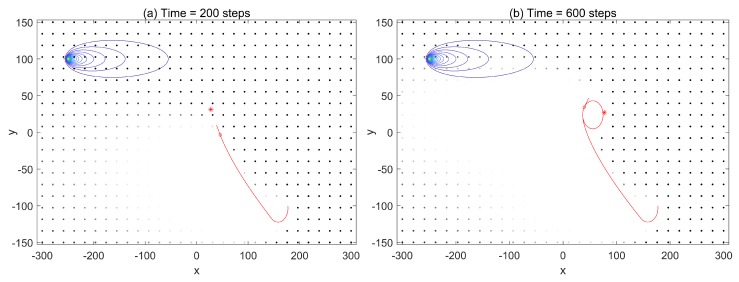
The trajectory (red line), detections (red solid circles), estimated source (red star), and particles (black dots) of the mobile sensor platform using the free energy scheme (T=0). The source location at (−250,100) with the contour plot of the corresponding mean rate.

Figure 6 shows that the search failed with setting the temperature T=0, and the sensor platform was eventually self-trapped around the estimated source, deviating from the real source. With the temperature T=0, the free energy only maintained the term of the potential energy. As a result, the sensor platform driven by the potential energy directly chased the estimated source. The probability distribution of the source was updated passively along the path approaching the estimated source. When the sensor reached the estimated source, the further update of the probability distribution of the source was not available (the expected source indicated by the red star hardly moved). In general, the exploitation driving the mobile sensor platform toward the most probable source is risky without a reliable estimation (requiring exploration to improve the reliability).

**Figure 7:**
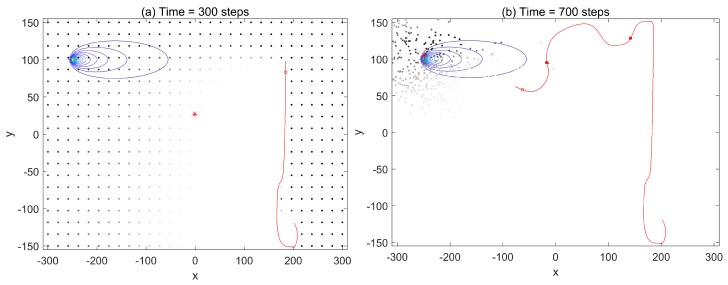
The trajectory (red line), detections (red solid circles), estimated source (red star), and particles (black dots) of the mobile sensor platform using the free energy scheme ($T=10^{4}$). The source location at (−250,100) with the contour plot of the corresponding mean rate.

Figure 7 shows that the search can be accomplished by the free energy infotaxis scheme with temperature $T=10^{4}$. As $T=10^{4}$ is big enough, the free energy was principally dominated by the term of entropy. The minimization of entropy drove the sensor to gather information and actively update the probability distribution of the source. Wherever the source was located, the sensor platform explored the space up to acquiring the positive detections to resample the particles. Thus, the mobile sensor platform was not trapped and kept improving the probability distribution.

To maintain the efficacy of the free energy infotaxis scheme starting with no prior knowledge about the space, the temperature *T* should make the value of entropy reduction dominate at the initial stage so that the sensor explores the workspace first. In general, the terms of exploitation and exploration should be combined and balanced in the search context. The exploration is principal to drive the search (gathering information and improving the estimation), and the exploitation can speed up the search progress. The potential energy and the entropy is unified in the free energy, and an adjusted temperature *T* actively controls the relative value between them.

## 5. Conclusions

This work deployed a mobile binary sensor platform to search for a diffusive source in turbulent flows. To solve the problem of the exploration-exploitation getting out of balance in the infotaxis scheme, we proposed a free energy infotaxis scheme that combines the potential energy and the entropy into free energy to be minimized as the reward of POMDP. The reduction of entropy maintains the role of exploration, which gathers information and increases the reliability of source estimation. The exploitation of chasing the most probable source location was carried out by the reduction of potential energy, which employed the weighted sum of the distance between all the hypothetical source locations and the sensor’s position. An adaptive internal temperature actively controlled the relative value between the potential energy and the entropy by leveraging the spread of the sampled particles measured by the trace of the covariance matrix. Thus, the exploitation-exploration balance was implemented by the fact that the exploration dominated the search in the stage with high uncertainty about the source, and then, the exploitation dominated the search with the receding of the uncertainty. The simulation results verified that the free energy infotaxis search scheme sped up the search for a diffusive source based on the sporadic binary detections. 

## Figures and Tables

**Table 1:** Mean search time (steps) for the infotaxis methods with varying scale of the search area.

Space Scale	100 × 100	150 × 150	200 × 200	250 × 250	300 × 300	350 × 350
infotaxis [14]	376.8	641.1	989.5	1156.9	1419.3	2136.5
the proposed method	335.7	483.6	821.1	993.5	1251.2	1982.0
mapless infotaxis [15]	347.9	535.2	864.5	1108.4	1391.3	2109.3
Infotaxis II [16]	372.1	659.2	917.5	1225.8	2389.9	3340.2
Infotaxis III [16]	375.4	646.7	928.0	1103.4	1535.4	2372.9
entrotaxis [17]	381.5	625.6	901.4	1157.8	1554.3	2269.5

## Abbreviations

The following abbreviations are used in this manuscript:

POMDP	Partially-Observed Markov Decision Process
RMIT	Royal Melbourne Institute of Technology
GMM	Gaussian Mixture Model
